# Estimation of optimal tilt angles for photovoltaic panels in Egypt with experimental verifications

**DOI:** 10.1038/s41598-023-30375-8

**Published:** 2023-02-25

**Authors:** Ashraf K. Abdelaal, Attia El-Fergany

**Affiliations:** 1 Department of Electrical Power and Machines, Suez University, Suez, 43512 Egypt; 2 Electrical Power and Machines Engineering Department, Faculty of Engineering, Zagazig University, Zagazig, 44519 Egypt

**Keywords:** Electrical and electronic engineering, Engineering

## Abstract

The principal target of this work is to compute the optimal tilt angle (OTA) for Photovoltaic (PV) panels. To perform this task, comprehensive simulations are done starting from altering the tilt angle (TA) daily, to use one fixed TA for all the year. The mathematical models for extra-terrestrial radiation (ETR) of both horizontal and inclined surfaces are presented firstly. At a later stage, the optimization formulation for the maximizing the solar radiation (SR) is adapted, and then the daily, monthly, seasonally, half-yearly and optimal fixed TAs are obtained. Although, the daily OTA produces the maximum SR, it is costly and impractical. It is found that altering the TA twice a year at optimal values that are computed as 5° and 50° for Suez city, gives the best results that are very near to the daily altering of the OTA. The difference between the two methods is 1.56% which is very small. Also, the two OTAs has SR better than that of the fixed OTA which is 28° by 7.77%. Also, it is found that the yearly fixed OTA (28°) is nearly equal to the latitude angle of Suez city which is 30°. The two OTAs method of this paper is different from the commonly used method that suggests two TAs. The first TA is used for winter months which is obtained by adding 15° to the latitude angle while the second TA is obtained by subtracting 15° from the latitude angle for the summer months. This commonly used method produces lesser SR than the two OTAs method of this paper. The theoretical work has been proved by an experimental work on two PV systems constructed at 25° and 30° TAs. The results of the experimental work agree with the theoretical results.

## Introduction

Due to the harmful effects of climate change, the world tries to reduce CO_2_ emissions and air pollution. The world has a strong desire to quickly move towards zero carbon emission energy sources such as renewable energies. In the last decade, utility scale solar PV systems are growing very fast, and their costs are continuing falling driving utilities to install more of these energy sources for electrical energy production. There are several variables disturbing the energy output of the PV panels^1–3^. One of these variables is the tilt or slope angle of the PV arrays. The TA is defined as the slope angle of the PV panel to the horizontal plane.

Many researchers were implemented in many countries to calculate the OTA. Zamora^4^ investigated the TA of the PV panel in areas with small values of latitude angles. A method is used by Agrawal and Chhajed^5^ in areas near the equator to enhance SR by 18.4%. A solar system is used by Shariah et al.^6^ to heat water in Jordon with TA varies from 0 to 20 depending on the city. A study was done by Osmani et al.^7^ to relate economics with the TA, in which they tried to reduce the price of energy and at the same time, reduces the installation costs. Liu et al.^8^ implemented a study in China to compute the TA by using measured data without giving any values for the OTA. Umunnakwe et al.^9^, research has been done to compute the OPT in many countries around the world; one of them is Sharm El-Sheikkh, Egypt with OTA of 23.2° which is agreed with the OTA presented in this paper. Karinka and Upadhyaya ^10^ introduced a method to compute the OPT and they conclude that 12.0% of energy reduction if the tilt angle is not varied each month, which contradicts with the outcomes of this paper. Abdallah et al.^11^ made a comparison between SR of Horizontal Surface (HS) with that of the tilted surface which is varied each month with different 15%, which is less than the SR of this paper which is 24%. Xu et al.^12^ suggested a technique to compute the OTA on hilly places.

Yadav et al.^13^ suggested a method to compute the OPT in the presence of houses shadow. Similar objectives could be found in^14–16^. Mansour et al.^17^, a similar study to get the OTA for different cities in Saudi Arabia. Tiris and Tiris in ^18^, Tiris and Tiris tried to compute the OTA of a collector system depending on the highest SR. Sarr et al.^19^, made a study to get the OTA in Senegal. They concluded that, the PV panel must be directed towards south in the north half of the earth, consequently the angle of azimuth is zero. Then the PV panels should be tilted to the horizontal by a TA. They mentioned that the OTA is not identical to the place latitude angle. Al-Sayyab et al.^20^ suggested that the TA in Basrah city (latitude 30) is 28 which agree with the result of this work. Chih-Chiang Wei^21^ presented evaluation models to forecast the sun energy radiation on tilted surfaces. In^22,23^ a simple correlation is presented to compute the mean monthly daily global radiation. Qasaimeh et al.^24^ implemented a study in Jordon to compute the OTAs and they concluded that their values are limited between 10° and 60°. Li et al.^25^ a mathematical method is presented to compute the SR on tilted surfaces by incorporating the measured sky radiation scattering. Then the yearly radiation at tilted collectors were determined. Islam et al.^26^ made a study to compute the OTA at Dhaka (latitude is 23.81°). It is found that the value of the OTA is 20.83° at Dhaka.

A more practical study is done by Benghanem^27^, in which he tried to calculate the OTA depending on measuring the values of the global daily SR and the diffuse SR on a flat surface. The OTA is computed, and it is nearly equivalent to the latitude angle. Also, they Clearfield that the OTA computed at each month has total radiation better than the optimum constant TA by 8%. Calabr^28^ proposed a technique to compute the OTA of PV system by using both the SR computed on horizontal plane and the data provided from weather stations. He concluded that the OTA can be computed from the latitude angle by a linear regression. Ahmad et al.^29^ analyzed the influence of the latitude angle on the working of sun trackers is tested and concluded that the performance of these sun tracked is highly dependent on the latitude angle. Alatarawneh et al.^30^ made a study to find out the importance of SR in Jordon and finding the OTA. While the latitude angle is 30.19°, the yearly optimal fixed TA is obtained to be 28.7°. The SR at the calculated optimal angle is higher than the SR at that of the latitude angle by 0.2%. They recommended that altering the panel angles four times a year which is not practical.

Khorasanizadeh and Mohammadi^31^ the OTA in the Iranian city of Tabass is determined. The yearly optimum tilt is 32°, while the latitude angle of Tabass is 33.36°. Jafarkazemi and Saadabadi^32^ computed the yearly OTA for Abu Dhabi city and its value was equal to 22° which was near to Abu Dhabi latitude 24.4°. Darhmaoui and Lahjouji^33^ showed that the difference between the experimental and the mathematical OTA is 8º which is too high. Kecili et al.^34^ Suggested many schemes for the OTA, and claimed that the number of variations of the TA increase, the SR will increase.

Salari and Javaran^35^, stated that 23% increase in SR in case of using fixed OTA| compared with the HS, which is overestimated. Ahmad and Tiwari^36^ presented a study to increase the SR on Horizontal dish, and claimed that the seasonal OTA is the best way to get highest SR. Many references claimed that the value of the TA has the same value as the latitude angle ± 15°. Where the positive sign is used in winter days and the negative sign is used in summer days. Similar work was done by Abed and Al-Salami^37^ in Turkey.

All the previous methods show some important facts as follows: (i) the importance of the TA for high efficiency of PV systems, (ii) the value of the OTA has a strong relation with the latitude angle, and (iii) the world is directed to use solar energy instead of fossil fuel.

Since Egypt has sunny days all over the year especially in the south. Egypt is directed to use solar energy and to build many plants around the country. One of these recommended areas is the Suez area that has high level of SR with very low clouds all the year and it is selected for this study beside three other cities to cover most of the Egyptian land. Also, Suez is near to the load center and has sunny climate inspires the Egyptian government and other international companies to build many PV systems there.

The main contributions of this work are: (i) If no TA is used, the PV system may lose a 95% of its daily SR or 24% of its annual SR, (ii) The two OTAs method of this paper is nearly equivalent to the daily altering of the OTA, (iii) One optimal fixed tilt can be used but its SR will be lesser by 7.01% than the two OTAs method, (iv) The fixed OTA is very near to the latitude angle, always lesser by 2°, and (v) Latitude angle can be used as a suboptimal TA with very small error.

This paper is organized as follow: the first part gives short review in the work done in the computation of the TA methods. The second part explains in short, the main governing equations to calculate the horizontal and tilted surfaces extraterrestrial sun radiation. Section “The importance of TA for PV system” explains the importance of the TA for optimizing the SR. Section “Problem formulation” puts the SR in a mathematical optimization form. Section “Simulation results” introduces 5 cases for computing OTAs. The last section displays the experiential work that has been implemented.

## Extraterrestrial SR relations

The following variables and parameters affect the solar energy radiation received by the PV arrays^38^.

### The solar constant $\left({{\varvec{G}}}_{{\varvec{s}}{\varvec{c}}}\right)$

The solar constant ${\mathrm{G}}_{\mathrm{sc}}$ is the value of the energy gained by the earth on one square meter. It has a value of 1353.0 W*/*m^2^ with an inaccuracy of ± 1.5%. ${\mathrm{G}}_{\mathrm{sc}}$ of value 1367.0 W*/*m^2^ is used in this paper.

### The earth-sun distance

Earth moves throughout the sun in an oval path. Therefore, the sun-earth distance changes from day to another. The mean distance between sun and earth is 150 Mkm. The quadratic of the reciprocal distance ratio between the earth and the sun, is given as ${\mathrm{E}}_{o}.$

1 $${\mathrm{E}}_{o}= ({\mathrm{r}}_{o}/\mathrm{r}{)}^{2}= 1.00011 + 0.034221\,\mathrm{ cos\,\mu }+ 0.00128\,\mathrm{ sin\,\mu }+ 0.000719\,\mathrm{ cos }\,2\upmu + 0.000077\,\mathrm{ sin }\,2\upmu$$

${\mathrm{E}}_{o}$ is termed as the eccentricity modification factor of the earth’s path^38^, and $\upmu$ is the angle cut by one day in radians.

### Solar declination (δ)

The earth moves throughout the polar axis, which has a slope of 23.5° from the equatorial plane. Meanwhile the vertex angle formed by the line connecting both earth and sun centers and the normal line to the center plane of the earth is known as the solar declination angle (δ). This angle changes every day. It has zero value at both spring equinox and autumnal equinox. δ is approximately equal to + 23.5° at the summer solstice and about − 23.5° at the winter solstice (for north half of the earth). The following expression for δ, in degrees^38^:

2 $$\updelta =(0.006918-0.399912\,\mathrm{cos\,\mu }+0.070257\,\mathrm{sin\,\mu }-0.006758\,\mathrm{cos}\,2\upmu + 0.000907\,\mathrm{sin}\,2\upmu - 0.002697\,\mathrm{cos}\,3\upmu + 0.00148\,\mathrm{sin}\,3\upmu )\left(\frac{180}{\pi }\right)$$

The hour angle (ω) is the angle measured at the celestial pole between the spectator’s meridian and the sun meridian. It is measured from mid-day, and it has 15° per one hour.

The sunrise hour angle $\left({\omega }_{s}\right)$ could be calculated as expressed in (3).

3 $${\omega }_{s}=co{s}^{-1} \left(-tan\,\varphi\,tan\,\delta \right)$$

Assuming that the PV panels are directed toward the equator, then the sunset hour angle is identical to the minus of the sunrise hour angle.

### The ETR on a horizontal surface

The daily SR outside the earth on a HS is given by the following equation:

4 $${H}_{o}=\left(\frac{24}{\pi }\right){I}_{SC} {E}_{o}\,\mathrm{sin}\,\varphi .\mathrm{sin}\,\delta .\left[\frac{\pi }{180} {\omega }_{s}-\mathrm{tan}\,{\omega }_{s}\right]$$

### The daily ETR on a tilted surface

The daily radiation outside the earth on a tilted surface is given by the following equation:

5 $${H}_{o\upbeta }=\frac{24}{\pi }{I}_{SC}{E}_{o}\left[\frac{\pi }{180} {\omega }_{s}^{^{\prime}}\,sin\,\delta\,\mathrm{ sin}\left(\varphi -\upbeta \right)+cos\,\delta\,\mathrm{ cos}\left(\varphi -\upbeta \right)sin\,{\omega }_{s}^{^{\prime}}\right], \forall {\omega }_{s}>{\omega }_{s}^{^{\prime}}$$

Or

6 $${H}_{o\upbeta }=\frac{24}{\pi }{I}_{SC}{E}_{o}\left[\frac{\pi }{180}\,{\omega }_{s}\,sin\,\delta\,\mathrm{ sin}\left(\varphi -\upbeta \right)+cos\,\delta\,\mathrm{ cos}\left(\varphi -\upbeta \right)sin\,{\omega }_{s}\right], \forall\,{\omega }_{s}\le {\omega }_{s}^{^{\prime}}$$

where

7 $${\omega }_{s}^{^{\prime}}=min\,\{{\omega }_{s}, co{s}^{-1} \left[-tan\,\delta\,tan\,\left(\varphi -\beta \right)\right]\}$$

### Sun path

**Figure 1:**
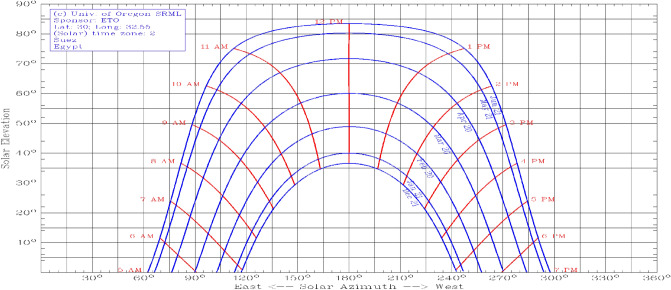
Sun-paths for Suez University.

The sun path is a picture of the sun's motion across the sky at specified location. With the help of the sun path, the azimuth and sun rise angles can be calculated. The sun path at Suez University (Latitude = 29.9987° and Longitude 32.5044°) is shown in Fig. 1 using the method described in^39^.

## The importance of TA for PV system

If someone is interested to get the maximum SR on a specified day (consider it as first of January) on Suez University area where the latitude is 29° 59′ 55.68ʺ N. According to that day the following has been calculated as φ = 29° 59′ 55.68ʺ = 29.9988° at Day number = 1 (first day in the year) as follows:

- Day Angle = $$\upmu =2\uppi \left(\mathrm{n}-1\right)/365$$ $$=$$ 360(1–1)/365 = 0,
- The earth-sun distance ratio is ${E}_{o}=( \frac{{r}_{o}}{r}{)}^{2}$ from Eq. (1), one can get E_o_ = (r_o_/r)^2^ = 1.0351,
- The declination angle δ is calculated using Eq. (2) and for January 1st δ = − 23.0558°,
- It should be remembered that both E_o_ and δ depends only on the day number and not in the TA. The sunrise angle is represented by Eq. (3) which gives ${\omega }_{s}=75.776{8}^{o}$, and
- The Daily Radiation value on a HS on that place is calculated from Eq. (4) or ${H}_{o}$=6.8974 kWh/ m^2^/day.

### The ETR on inclined surface directed to south

The daily ETR on a inclined surface directed to the south can be calculated from Eqs. (5) or (6), and the value of ${\upomega }_{\mathrm{s}}^{\mathrm{^{\prime}}}$ can be calculated from Eq. (7) as ${\upomega }_{\mathrm{s}}^{\mathrm{^{\prime}}}=\mathrm{min }\{75.7768,\mathrm{ co}{\mathrm{s}}^{-1} \left[-\mathrm{tan\,\delta\,tan }\left(\mathrm{\varphi }-\upbeta \right)\right]\}$ . 

**Figure 2:**
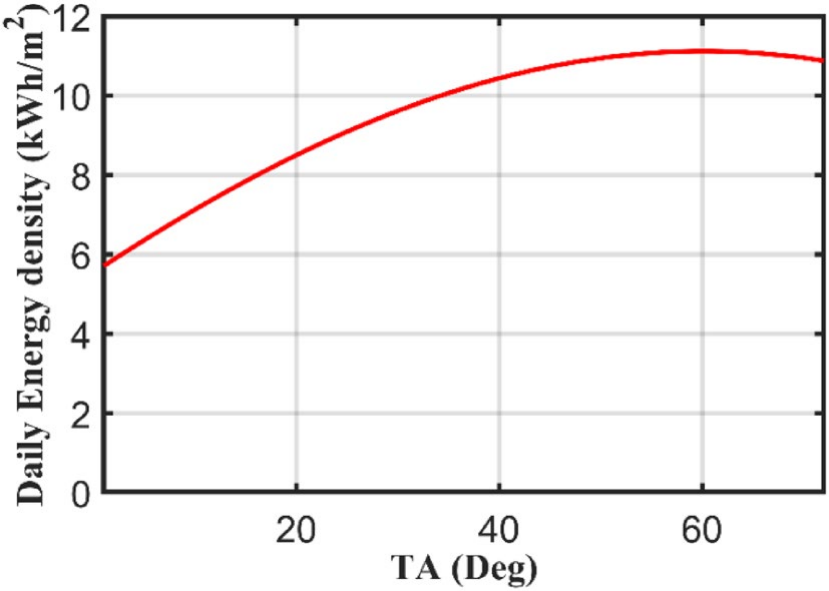
The influence of TA variation on extraterrestrial solar radiation.

If Eqs. (5) or (6) is simulated using MATLAB for dissimilar TAs varying from 0° to 90°, for a specific day for example 1st of January, the following curve shown in Fig. 2 is obtained. The maximum radiation at January first on a tilted surface is 11.1167 kWh and is obtained at TA of 60°. The radiation is increased from 5.7011 kWh at zero TA to 11.1167 kWh at OTA for that day of 60°. The percentage increase in radiation is 95% which indicates the significance of the TA.

**Figure 3:**
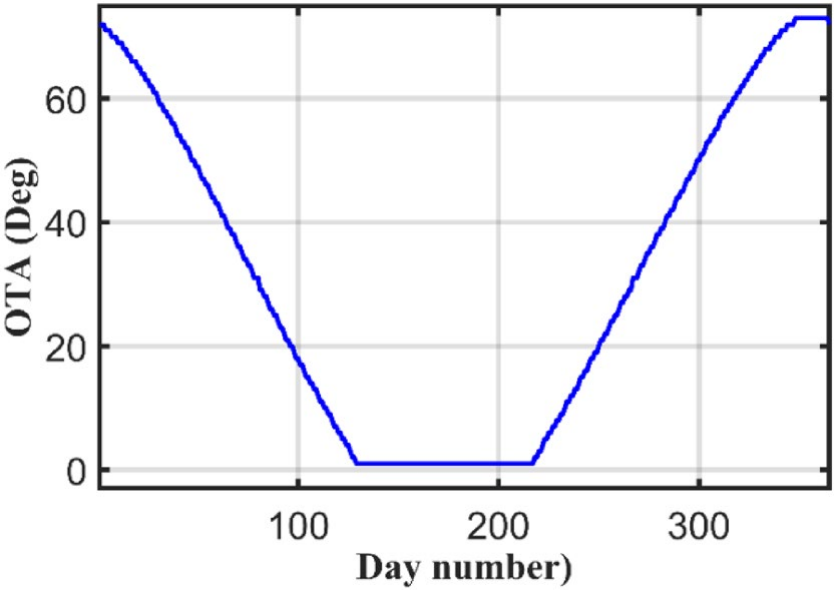
Optimal values of daily TAs.

Another important question is that how many times should the TA to be changed per year? Should the TA to be changed 365 times per year (daily), 12 times per year (monthly), 4 times per year (seasonally), 2 times per year or fixed at suboptimal angle? To answer this question, the maximum total radiation is the main factor for selecting the OTA. A simulation has been made for all days in the year for TAs from 0° to 90° and the simulation results are given in Fig. 3 which displays the optimum daily TA for all days of the year for Suez city (Latitude 29.9988° ≈ 30°). According to Fig. 1, one can select how many times that can be used for the TA along the year.

The curve has symmetry around June month. Also, one can notice that the year can be distributed into three intervals. The first interval is the winter season, starting from 1st of January to 28 of February, of total 59 days. The key feature of this interval is that the OTA is decreasing during January and February, while it is increasing during November and December. Based on the previous discussion an optimum TA of 50° will be chosen for that interval. A closer look to Fig. 3, the reader can notice that during summer months from May to August, the TA is 1°. This can be explained using the sun path curves shown in Fig. 1 for these months. The sun paths during these months have flatted top as shown in Fig. 1 for many hours, so the sun rays are nearly normal to the earth during these hours. So, to harness more radiation it is better to make the PV panels nearly horizontal or tilted at very small angle. For these months, the OTA is 1°.

## Problem formulation

The main goal is to get the highest yearly solar radiation. So, our objective function can be adapted to maximize the yearly total radiation as per Eqs. (5) and (6). This function has discontinuity at ${\omega }_{s}$ since the condition ${\omega }_{s}^{^{\prime}}\le {\omega }_{s}$ in the objective function, and subject to the following constraints as depicted in Eqs. (8) to (13).

8 $$\upmu =2\uppi \left(\mathrm{n}-1\right)/365$$

9 $${E}_{o}=1.00011+0.034221\,\mathrm{cos\,\mu }+0.00128\,\mathrm{sin\,\mu }+0.000719\,\mathrm{cos}\,2\upmu +0.000077\,\mathrm{sin}\,2\upmu$$

10 $$\updelta =\left(0.006918-0.399912\,\mathrm{cos\,\mu }+0.070257\,\mathrm{sin\,\mu }-0.006758\,\mathrm{cos}\,2\upmu + 0.000907\,\mathrm{sin}\,2\upmu - 0.002697\,\mathrm{cos}\,3\upmu + 0.00148\,\mathrm{sin}\,3\upmu \right)\left(\frac{180}{\pi }\right)$$

11 $${\omega }_{s}={\mathrm{cos}}^{-1}(-\mathrm{tan}\,\varphi *\mathrm{tan}\,\delta )$$

12 $${\omega }_{s}^{^{\prime}}=\mathrm{min}\left\{{\omega }_{s}, co{s}^{-1} \left[-tan\,\delta *tan\,\left(\varphi -\beta \right)\right]\right\}$$

13 $$0\le \beta \le {90}^{o}$$

**Figure 4:**
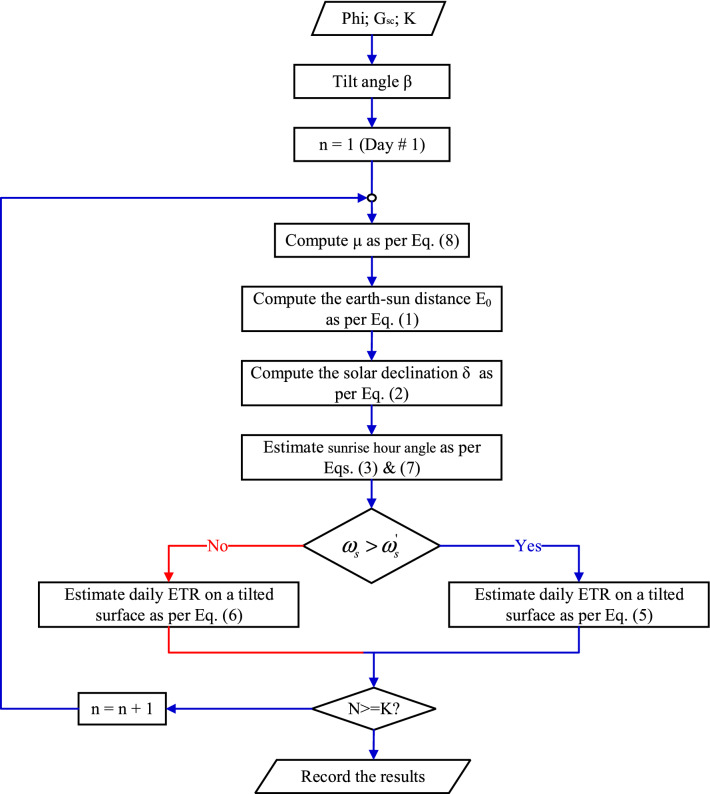
Computation procedure to calculate the SR for certain tilts angle β.

**Figure 5:**
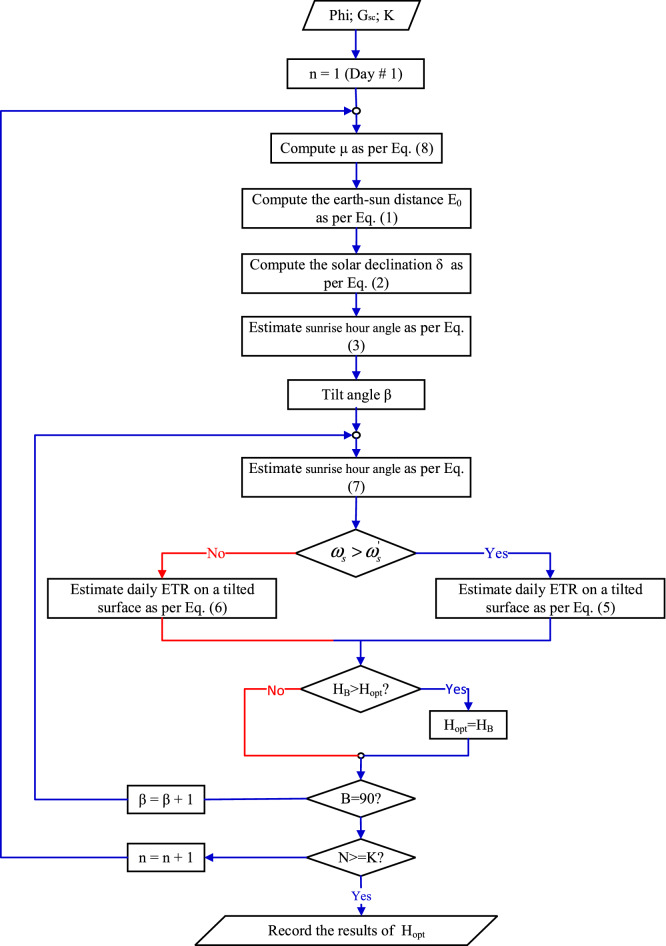
Computation of the OTA β for a specified interval K.

This is an optimization problem, but unfortunately it has two difficulties the first is the discontinuity in the objective function which is depends on the TA, and the second difficulty is the summing in the objective function. The flow chart in Fig. 4 shows the computation procedure to calculate the SR for certain tilt angle β for interval K. While the flow chart displayed in Fig. 5 illustrate the process to obtain the OTA β for a specified interval K.

## Simulation results

In this section, many scenarios are anticipated for further demonstration to validate the proposed approach. Based on both Figs. 1 and 3, the following scenarios are considered:

1. Zero TA or horizontal panel.
2. Daily altering of the TA of the panel.
3. Monthly altering of the TA of the panel.
4. Seasonal altering of the TA of the panel.
5. Two times altering of the TA of the panel.
6. Fixed TA.

It must be remembered that the TA changes will add additional cost to the PV systems costs. The optimization problem has been solved by a program written in MATLAB. The coming subsections detailed the analysis, discussions, and comprehensive results.

### Zero TA or horizontal PV panel

The ETR at HS or zero TA is 3204.5 kWh/ m^2^/ year which is calculated for Suez city.

### Daily altering of the TA

**Figure 6:**
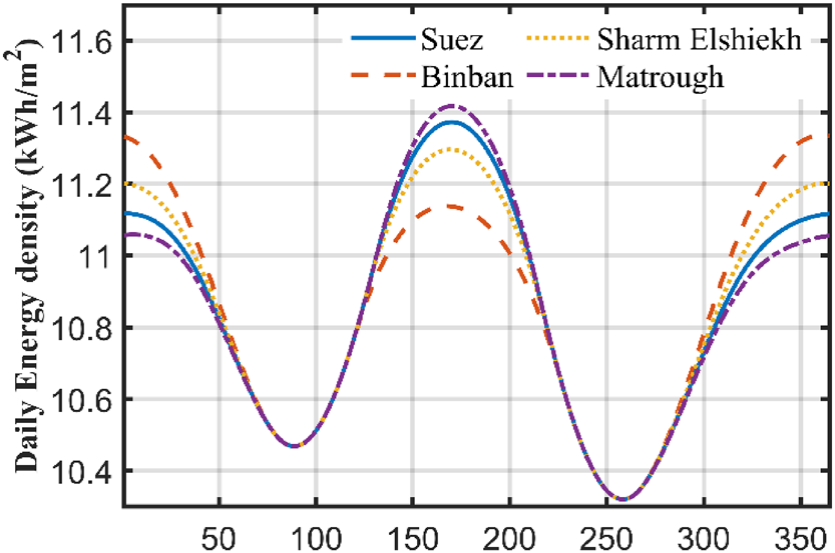
Solar radiation versus optimal daily TA for some cities in Egypt.

If the PV scheme is tilted daily at an OTA, the SR will be changed according to each day OTA. Figure 6 displays the total yearly extraterrestrial SR at daily OTA for four cities. The yearly SR for Suez city is 3974.6 kWh/ m^2^/year with a daily average value of 10.8894 kWh/day/m^2^. The curve is simulated by calculating the daily radiation for each TA starting from 1° to 90°, and then the angle of maximum radiation is obtained. This case is higher than the radiation of HS by 770.1 kWh in a *year or 24%*. This can save money. Some interesting notes can be noticed from Fig. 4, for example the maximum daily radiation in all the year is obtained at day number 172 (21st of June) with corresponding OTA of 1°. This can be explained by remembering that this day is the longest day in the year with sun shining for more than 14 h. Table 1 shows the OTAs with their corresponding total radiation at certain days in the year for 4 different places in Egypt. These places are chosen to cover most of the Egyptian country. The radiations at these cities are shown in Fig. 6.

**Table 1:** Optimal daily angle and radiation.

City	Suez		Binban		Sharm Elshiekh		Marsa Matrough	
Day number	OTA Deg	Radiation kWh/m^2^	OTA Deg	Radiation kWh/m^2^	OTA Deg	Radiation kWh/m^2^	OTA Deg	Radiation kWh/m^2^
1	60	11.148	55	11.364	58	11.23	61	11.087
17	58	11.127	53	11.297	56	11.19	59	11.079
45	49	10.92	44	10.975	47	10.94	50	10.9
74	34	10.57	28	10.57	32	10.57	35	10.57
105	15	10.586	9	10.586	13	10.586	16	10.586
135	1	11.1	1	11.015	1	11.08	1	11.108
161	1	11.385	1	11.164	1	11.31	1	11.43
199	1	11.21	1	11.045	1	11.159	1	11.236
230	8	10.616	3	10.616	6	10.616	10	10.616
261	27	10.35	21	10.352	25	10.35	28	10.352
292	44	10.65	39	10.68	42	10.664	45	10.644
322	56	10.999	51	11.135	54	11.053	57	10.96
347	60	11.122	55	11.338	58	11.206	61	11.06
Yearly total		3963.52		3966.69		3966.12		3960.89

The difference between the four cities is very small less than 6 kWh in the whole year with Binban has maximum at 3966.69 kWh/m^2^/year and Matrough has the minimum at 3960.89 kWh/m^2^/year. Sues has maximum yearly radiation at 3963.52 kWh/m^2^/year. So, this means that most of the Egyptian cities have a high radiation level can reach 3960 kWh/m^2^/year.

### Monthly altering of the TA (12 times in a year)

**Figure 7:**
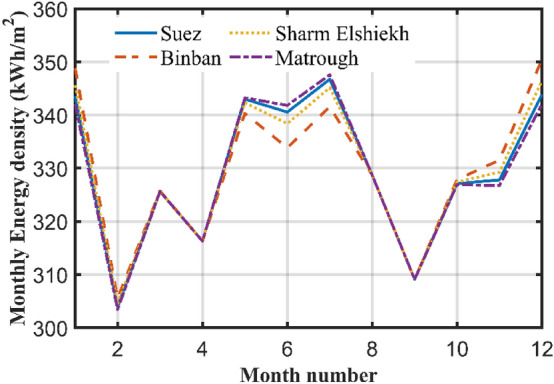
Solar radiation at monthly OTA for some cities in Egypt.

Since daily changing of the TA is difficult, costly, and it may be impractical to be implemented for PV panels, it is better to reduce the number of changing of the TA. In the present case, the TA is changed every month. The optimal monthly TA is computed depending on the monthly maximum sun radiation. For each month the tilt is changed from 0 to 90 degree, and then the angle of maximum radiation is determined. Table 2 displays the simulation results obtained for the present case in the same cities. For every month the OTA is calculated for the cities. Also, both the monthly total radiation and the daily average radiation are computed. The results are drawn in Fig. 7 which shows the total radiation for the four cities. The radiations for these cities are very near to each other. By comparing the total yearly radiation for this case and case two, one can discover that both cases have a near values. In the first case the yearly total SR in Suez city is 3963.52 kWh/m^2^, which is very near to the second case which is 3956.28 kWh. The total yearly radiation in this case is less than the first case by 7.24 kWh/m^2^ in all the year or 0.183% which is very small. This case shows a very important fact that varying the TA 12 times in a year is nearly equivalent to changing the TA 365 times or daily, thus no need to change the TA daily. Table 2 has been drawn in Fig. 7 which shows the monthly radiation for the four cities at monthly optimal angle.

**Table 2:** Monthly radiation at monthly optimal angle.

City	Suez			Binban (Aswan)			Sharm-Elshiekh			Marsa Matrough		
Month	β_opt_	Monthly rad kWh/m^2^	Av. daily Rad	β_opt_	Monthly rad kWh/m^2^	Av. daily Rad	β_opt_	Monthly rad kWh/m^2^	Av. daily Rad	β_opt_	Monthly rad kWh/m^2^	Av. daily rad
Jan	58	343.55	11.082	53	348.823	11.252	56	345.618	11.149	59	342.074	11.035
Feb	48	303.9	10.854	43	305.54	10.912	47	304.545	10.877	50	303.445	10.837
March	33	325.67	10.51	28	325.76	10.508	31	325.711	10.507	34	325.631	10.504
April	15	316.3	10.54	9	316.3	10.543	13	316.3	10.543	16	316.304	10.543
May	1	342.995	11.064	1	340.3	10.977	1	342.33	11.043	1	343.235	11.072
June	1	340.556	11.352	1	333.84	11.128	1	338.39	11.28	1	341.813	11.394
July	1	346.715	11.184	1	341.56	11.018	1	345.137	11.133	1	347.571	11.212
August	7	328.716	10.604	2	328.72	10.604	5	328.717	10.604	9	328.723	10.604
Sept	25	309.115	10.304	20	309.11	10.303	23	309.116	10.304	26	309.105	10.304
Oct	42	327.115	10.552	37	327.868	10.576	41	327.409	10.562	44	326.912	10.546
Nov	55	327.8	10.927	50	331.522	11.05	53	329.264	10.976	56	326.757	10.892
Dec	60	343.85	11.092	55	350.46	11.305	58	346.437	11.175	61	342.006	11.032
Total												
Case 3		3956.28			3959.80			3958.97			3953.58	
Case 2		3963.52			3966.69			3966.12			3960.89

### Variation of the TA 3 times per year (seasonally)

In this case the TA will be changed according to the season i.e. winter, spring, summer, and autumn. The first interval is the winter interval starting from 21st December to 20 of March of total 89 days. For Suez city, the OTA for this interval is computed in the same manner as in case 1 and 2, and it is found to be 51°, with total radiation in this period of 959.951 kWh/m^2^ and with daily average of 10.786 kWh/m^2^. The second interval includes spring days starting from 21st of March to 20 of June of total 93 days. The OTA is 6° with total SR of 993.634 kWh/m^2^ with daily average of 10.684 kWh/m^2^/day. In Summer season which starts from 22 June to 22 September, the OTA is 5° which is nearly identical to the spring season with total SR of 1000 kwh/m^2^ in 94 days with daily average of 10.638 kWh/m^2^/day. In Autumn which starts from 23 September till 20 December, the OTA is found to be equal to 50° which is also nearly equal to the winter season with total SR of 948.059 kWh/m^2^ in 89 days with daily average of 10.652 kWh/m^2^/day. The optimum angle and SR for the other cities are shown in Table 3.

**Table 3:** The optimal angle and solar radiation for Case 3.

Season	No. of days	Suez 30°		Binban 24.44°		Shram-elsheikh 27.9654°		Matrough 31.3543°	
		${\upbeta }_{\mathrm{opt}}$	Radiation (kWh/m^2^/year)	${\upbeta }_{\mathrm{opt}}$	Radiation (kWh/m^2^/year)	${\upbeta }_{\mathrm{opt}}$	Radiation (kWh/m^2^/year)	${\upbeta }_{\mathrm{opt}}$	Radiation (kWh/m^2^/year)
Winter	89	51	971.1024	47	979.90	49	974.6479	54	968.0599
Spring	93	6	993.634	1	993.6	4	993.635	7	993.612
Summer	94	5	1000	1	999.661	3	1000	6	1000
Autumn	89	50	948.059	45	955.9963	48	951.1984	51	945.813
Total	365		3912.795		3929.16		3919.48		3907.4849

By comparing the total yearly radiation of value 3901.6 kWh/m^2^ with case 2 that has a total yearly radiation of 3963.52 kWh/m^2^/year, one can notice that the difference is equal to 61.92 kWh/m^2^/year or + 1.56%. By comparison with the case three which has a value of 3956.28 kWh/m^2^/year the difference is 54.68 kWh/m^2^/year or + 1.38% which is small.

The most important outcome of this case is that, firstly varying the TA 4 times a year has a nearly equivalent to altering the TA 365 times a year or 12 times a year with an estimated error of 1.5%. The second outcomes can be noticed by checking the value of the OTA. From Table 3 one can notices that the winter OTA (which is 51° in winter) is approximately equal to the autumn OTA (which is 50° in autumn). So, one can use one TA for both autumn and winter seasons. Equal explanation can be used for spring and summer seasons. The result is only two OTAs are needed for the whole year with excellent accuracy.

### Variation of the TA 2 times yearly

From the results obtained in case 3, one concludes that is only two OTAs are enough, since both the winter and autumn OTAs are very near, and both the spring and summer OTAs are very near. So, the year can be divided into two interval one for autumn and winter starting from 23rd of September till 20 of March of total of 178 days and the second interval include both spring and summer of total of 187 days starting from 20 of March to 22nd of September. By simulation these intervals for Suez city, one can find that the first interval has an optimal value of 50° and the other TA is 5°. The radiation is 3901.72 kWh/m^2^/year which is less than the second case by 61.8 kWh/m^2^/year or 1.56% in all the year which is very small. By comparing this case with case 3, one can see that this case is very near to case 3. So instead of changing the TA four times a year, it is easier and more economical to change it two times a year. Table 4 summarizes the OTA and radiation for the four cities.

**Table 4:** Case 4: two optimal angles for the year.

Season	No. of days	Suez		Binban		Shram-elsheikh		Matrough	
		${\upbeta }_{\mathrm{opt}}$	Radiation (kWh/m^2^/year)	${\upbeta }_{\mathrm{opt}}$	Radiation (kWh/m^2^/year)	${\upbeta }_{\mathrm{opt}}$	Radiation (kWh/m^2^/year)	${\upbeta }_{\mathrm{opt}}$	Radiation (kWh/m^2^/year)
Winter + autumn	178	50	1908.26	46	1925.06	48	1914.916	51	1903.53
Summer + spring	187	5	1993.6	1	1993.3	3	1993.6	7	1993.6
Total	365		3901.86		3918.36		3908.52		3897.13

Comparing this case of two OTA with the commonly used two tilted angle method mentioned in several literature, which is using one TA in autumn and winter equal to Latitude angle + 15° and the other angle is equal to the Latitude angle − 15° in spring and summer. The optimal two angles of this case have a radiation of 3901.72 kWh/m^2^/year, while the common method has radiation of 3868.476 kWh/m^2^/year. As a result, this case is better with 3901.72–3868.476 = 33.2 kWh/m^2^/year which means + 0.86% higher than the two tilted angle method.

### Fixed TA

**Figure 8:**
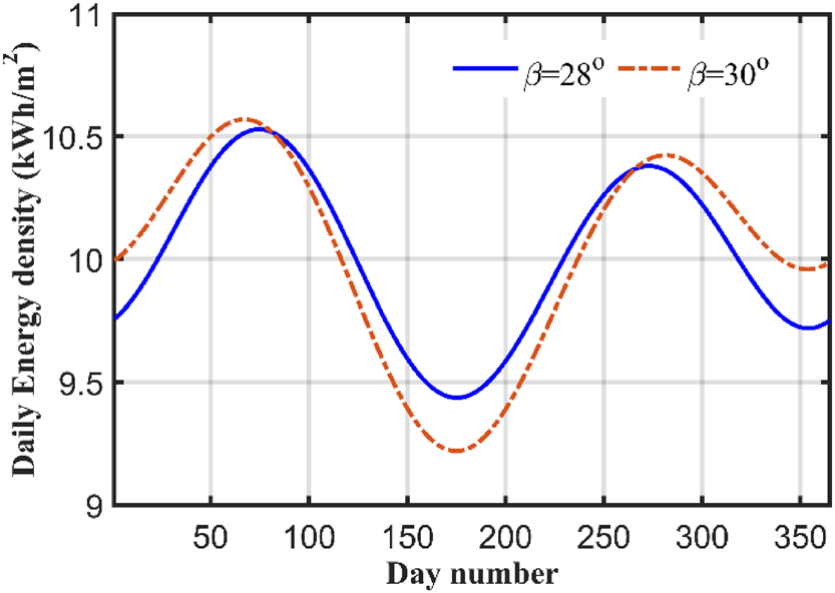
Comparisons between the radiation at OTA and the latitude angle.

In this case, the TA is kept fixed at an optimal yearly value. The OTA is calculated for Suez city and its value is equal to 28°, which is near to the latitude value. This is very common in practice to consider that the TA is identical to the latitude value. The total radiation in this case is 3620.3 kWh/m^2^/year, with an average daily of 9.9187 kWh/m^2^/day. Comparing this case with case 4, the difference between these two cases is 3901.72–3620.3 = 256.62 kWh/m^2^/year, or 7.77% which is not small. Comparing radiation computed at the OTA with the value of radiation computed at latitude angle which is 3619.2 kWh/m^2^/year. The radiation at the latitude TA is smaller than the radiation at the yearly OTA by 0.74% which is very small. Figure 8 shows the difference between the radiations at the two TAs.

Table 5 shows that the optimal yearly TA is very near to the latitude angle. The error between the two angles is very small and it is less than 0.03% and can be neglected from which, one can discover that the latitude angle can be used as a sub-OTA.

**Table 5:** Shows the OTA for other cities in Egypt.

City	Suez	Binban	Shram-Elshiekh	Marsa-Matrough
Latitude	30°	24.44°	27.9654°	31.3543°
OTA	28°	23°	27°	30°
Yearly radiation kWh (optimal)	3620.3	3630.3	3624.3	3617.4
Yearly radiation kWh (latitude)	3619.2	3629.5	3623.4	3616.0
Difference between optimal and latitude	0.03%	0.022%	0.0248%	0.0387%

## Experimental work

**Figure 9:**
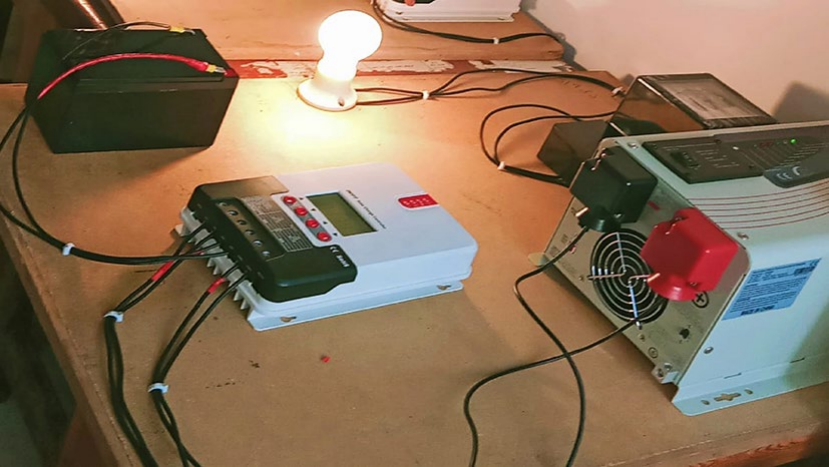
The system components and experimental setup for 25° TA.

**Figure 10:**
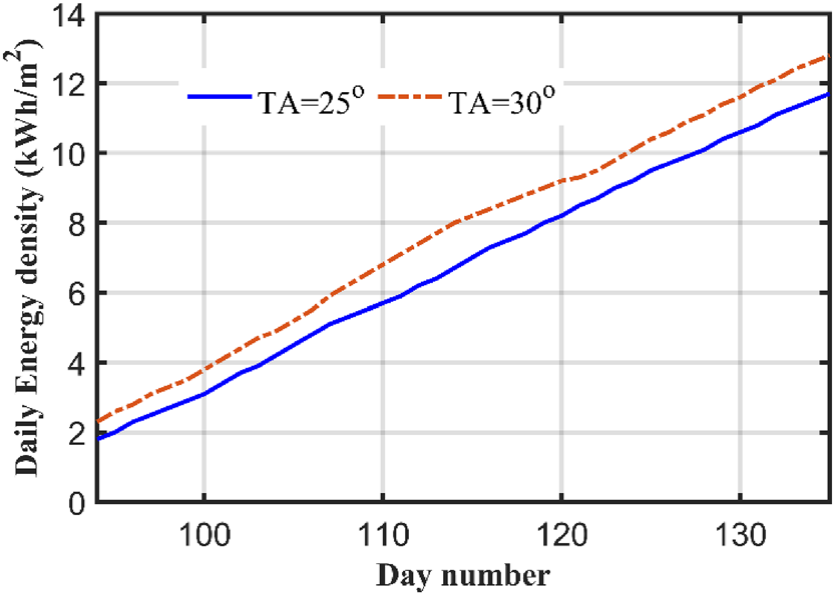
Energy consumed by both systems during the period starting from 4th of April to 30 of May.

For experimental verification for the theoretical work, two PV systems directed towards south, with two TAs of 25°, and 30°, are constructed in Suez University (latitude 30°). Each system has 100 W PV panel, maximum power point tracker, inverter, battery, and load. The complete architecture of one scheme is displayed in Fig. 9. The measurements are recorded daily for 57 days, starting from 4th of April till 30 of May. Some of the measurements are shown in Table 6 which shows the actual meter pictures in columns 3 and 5 for the 25° and 30° TAs systems respectively. These reading are rewritten in numbers in columns 4 and 6 for both systems. The measurements are shown in Fig. 10 which shows that the energy consumed for both systems in exactly 57 days which are shown in the horizontal axis by day number starting from the fourth of April (or day number 94) and ending at day 30 of May (or day number 150). In this period, the net energy consumed by the 25° TA system is equal to 13.9 kWh, with average daily consumption of 0.243 kWh. The 30° systems consumed energy of 13.3 kWh with average daily energy of 0.233 kWh/m^2^/day. The 25° TA is higher the 30° by an energy of 0.6 kWh or 4.4%. This agrees with the theoretical results obtained in case 2 which showed that the TAs in these months which are 15° (not very far from 25°) for April and 1° (which is very far from 30°) for May. Also from Fig. 8 and for months April and May, one can easily notice that the SR in these two months for 25° TL is higher than that of the 30°. Figure 10 shows this result with a small difference between the two systems.

**Table 6:** Readings of the two TAs PV systems.

Date	Temp. (°C)	25° TA system		30° TA system	
		Meter reading	Reading	Meter reading	Reading
4/4	36		2.3		1.8
10/4	36		3.8		3.1
16/4	35		5.5		4.8
20/4	36		6.8		5.7
26/4	35		8.4		7.3
28/4	36		7.7		8.8
7/5	36		10.9		9.9
10/5	38		11.6		10.6
15/5	38		12.8		11.7
19/5	38		13.6		12.6
25/5	40		15.0		13.9
30/5	39		16.2		15.1

### Human and animal rights

This article does not contain any studies with animals performed by any of the authors.

## Conclusions

This paper investigates the optimum TA for PV panels. The OTA for each day is calculated for some cities in Egypt. Although the optimum daily TA produces the highest SR value, but it may not be practical for PV panels. It can be used in some other application for example solar cookers. Also, both the optimal monthly and seasonal TAs are calculated for these cities in Egypt. After that the optimal yearly tow TAs are calculated for the whole year. The final case calculates one OTA for all the year which is very close to the latitude angle. From the results it has been shown that, although, the optimal daily angles give the best solar radiation, but it is not practical for PV panels. For practical standpoint, the best way is to use the two OTAs in the year since it gives a SR that is very near to the daily OTAs. Comparing the two OTAs presented here with the commonly used method which use a TAs equal to Latitude angle + 15° for autumn and winter months and the other angle is the Latitude angle – 15° for spring and summer months. When simulating this method, the two OTAs presented in this paper calculated at TAs of 5° for spring and summer months and 50° for winter months for Suez city, this method gives SR of 3901.72 kWh/year/m^2^ which is higher that the commonly used two TAs method. A fixed TA can be used, but the SR will be lower than the two optimal angels’ method. An experimental work has been done on two small PV systems, the first one is at a TA of 30° (latitude angle of Suez), the other value of TA is 25°. Measurements have been recorded during April and May. The Measurements show that for that period the TA of 25° is better than of the 30° TA by 4.4% which is coincidence with the theoretical results.

## Data Availability

The data that support the findings of this study are available from the corresponding author upon reasonable request.
